# D-Tryptophan Promotes Skin Wound Healing via Extracellular Matrix Remodeling in Normal and Diabetic Models

**DOI:** 10.3390/ijms26157158

**Published:** 2025-07-24

**Authors:** Dawit Adisu Tadese, James Mwangi, Brenda B. Michira, Yi Wang, Kaixun Cao, Min Yang, Mehwish Khalid, Ziyi Wang, Qiumin Lu, Ren Lai

**Affiliations:** 1 Key Laboratory of Genetic Evolution & Animal Models, Engineering Laboratory of Peptides of Chinese Academy of Sciences, Key Laboratory of Bioactive Peptides of Yunnan Province, KIZ-CUHK Joint Laboratory of Bioresources and Molecular Research in Common Diseases, National Resource Center for Non-Human Primates, and Sino-African Joint Research Center, New Cornerstone Science Laboratory, Kunming Institute of Zoology, The Chinese Academy of Sciences, Kunming 650201, China; davadisu@gmail.com (D.A.T.); jams@mail.kiz.ac.cn (J.M.); brenda@mail.kiz.ac.cn (B.B.M.); yangmin@mail.kiz.ac.cn (M.Y.); mehwishkhalidd120@gmail.com (M.K.); wangziyi@mail.kiz.ac.cn (Z.W.); lvqm@mail.kiz.ac.cn (Q.L.); 2 University of Chinese Academy of Sciences, Beijing 100049, China; 3 Kunming College of Life Science, University of Chinese Academy of Sciences, Beijing 100049, China; 4 Center for Evolution and Conservation Biology, Southern Marine Science and Engineering Guangdong Laboratory (Guangzhou), Guangzhou 511458, China; 13477288321@163.com; 5 College of Life Sciences, Nanjing Agricultural University, Nanjing 210095, China; kaixuncao@gmail.com

**Keywords:** D-tryptophan, diabetic wound healing, hypoxia-inducible factor 1-alpha, extracellular matrix, inflammation

## Abstract

Diabetic wounds are a devastating complication that cause chronic pain, recurrent infections, and limb amputations due to impaired healing. Despite advances in wound care, existing therapies often fail to address the underlying molecular dysregulation, highlighting the need for innovative and safe therapeutic approaches. Among these, D-amino acids such as D-tryptophan (D-Trp) have emerged as key regulators of cellular processes; however, their therapeutic potential in diabetic wounds remains largely unexplored. Here, we investigate the therapeutic potential of D-Trp in streptozotocin (STZ)-induced diabetic mice, comparing it with phosphate-buffered saline (PBS) controls and vascular endothelial growth factor (VEGF) as a positive control. Wound healing, inflammation, and histopathology were assessed. Protein and gene expression were analyzed via Western blot and RT-qPCR, respectively. Biolayer interferometry (BLI) measured the binding of D-Trp to hypoxia-inducible factor-1α (HIF-1α). D-Trp accelerated wound healing by modulating extracellular matrix (ECM) remodeling, signaling, and apoptosis. It upregulated matrix metalloproteinases (MMP1, MMP3, MMP-9), Janus kinase 2 (JAK2), and mitogen-activated protein kinase (MAPK) proteins while reducing pro-inflammatory cytokines (tumor necrosis factor-α [*TNF-α*], interleukin-1β [*IL-1β*], *IL-6*). D-Trp also suppressed caspase-3 and enhanced angiogenesis through HIF-1α activation. These findings suggest that D-Trp promotes healing by boosting ECM turnover, reducing inflammation, and activating MAPK/JAK pathways. Thus, D-Trp is a promising therapeutic for diabetic wounds.

## 1. Introduction

Wound healing is an intricate and dynamic process crucial for restoring tissue integrity and function following injury or skin damage [1]. This complex biological process involves a meticulously coordinated sequence of steps that aim to repair injured tissue, restore the skin barrier, and achieve complete tissue regeneration [2]. Rapid and efficient wound healing of skin is critical for homeostasis, preventing infections, and restoring the structural and functional integrity of the skin, the largest organ of body [1]. There are several discrete but interrelated phases of wound healing, including hemostasis, inflammation, proliferation, and remodeling [3]. Each phase is defined by distinct cellular and molecular occurrences controlled by several signaling pathways, growth factors, cytokines, and immune cells [4]. Impaired wound healing, especially in individuals with chronic diseases such as type 2 diabetes, poses a significant clinical challenge [5,6], resulting in delayed closure [7], increased risk of infection [8], and prolonged consequences [9]. Comprehending the complex principles of wound healing is crucial for formulating efficient therapeutic techniques to improve the healing process and increase tissue restoration results.

Diabetic wounds, a common and serious complication of diabetes mellitus (DM), can cause serious problems such as amputation and infection-related death, primarily due to poor circulation and sensory loss on the feet [10]. Refractory diabetic foot unclears (DFUs) are particularly concerning, as they frequently cause non-traumatic amputations and are a major health risk for diabetics [11,12]. Delayed wound healing is often due to chemokine deficiency, abnormal inflammation, and insufficient angiogenesis and epithelial regeneration [13,14]. Current treatments, such as wound debridement, anti-inflammatory medications, and topical antibiotics or growth factors, offer limited effectiveness [2]. Examining diabetic wounds from various perspectives could help identify new targets and strategies for treating refractory diabetic wounds [15,16,17,18,19,20,21]. Likewise, advances in wound healing research, including stem cell therapies, tissue engineering methods, and personalized medicine strategies, have significant potential to transform the wound care domain [22]. Researchers seek to develop innovative therapies that enhance healing speed, minimize scarring, and improve patient quality of life by elucidating the molecular mechanisms underlying wound healing, identifying novel therapeutic targets, and leveraging the regenerative capabilities of stem cells [23,24,25].

D-amino acids, the enantiomers of L-amino acids, were historically considered to be non-functional. However, recent studies have demonstrated that certain D-amino acids present in mammalian tissues play a significant role in various physiological processes [26]. Among these, D-Trp has gained attention for its diverse metabolic functions and potential therapeutic applications. A separate study reports that D-Trp suppresses colitis progression by reducing specific intestinal microorganisms. Moreover, D-Trp augments intracellular levels of indole acrylic acid (IA), a critical molecule that modulates the susceptibility of enteric microorganisms to D-trp. Administration of IA has been shown to improve the survival rates of mice infected with *C. rodentium* [27]. Moreover, research has demonstrated that D-Trp can reduce the initial adhesion of pathogenic cells and alter the extracellular environment, leading to a notable decrease in pathogen viability [28]. Additionally, D-Trp inhibits the formation of biofilms through similar mechanisms and acts as an auto-inhibitory compound that prevents the germination of spores and the growth of harmful microorganisms [27,28,29,30]. Extensive studies have been conducted on the interaction between signaling pathways and important proteins in wound healing [31]; however, the potential therapeutic role of D-Trp, as an amino acid, remains largely unexplored. Thus, we aimed to evaluate the therapeutic efficacy of D-Trp in improving extracellular matrix (ECM) remodeling and facilitating wound closure in both normal and diabetic models. The findings underscore D-Trp as a potentially effective metabolite-based therapeutic agent for the promotion of wound healing. This research lays the foundation for the advancement of innovative metabolite-driven treatments that offer not only enhanced efficacy, but also a favorable safety profile, representing a significant advancement in wound care.

## 2. Results

### 2.1. D-Trp Promotes the Migration of HaCaT Cells

**Figure 1:**
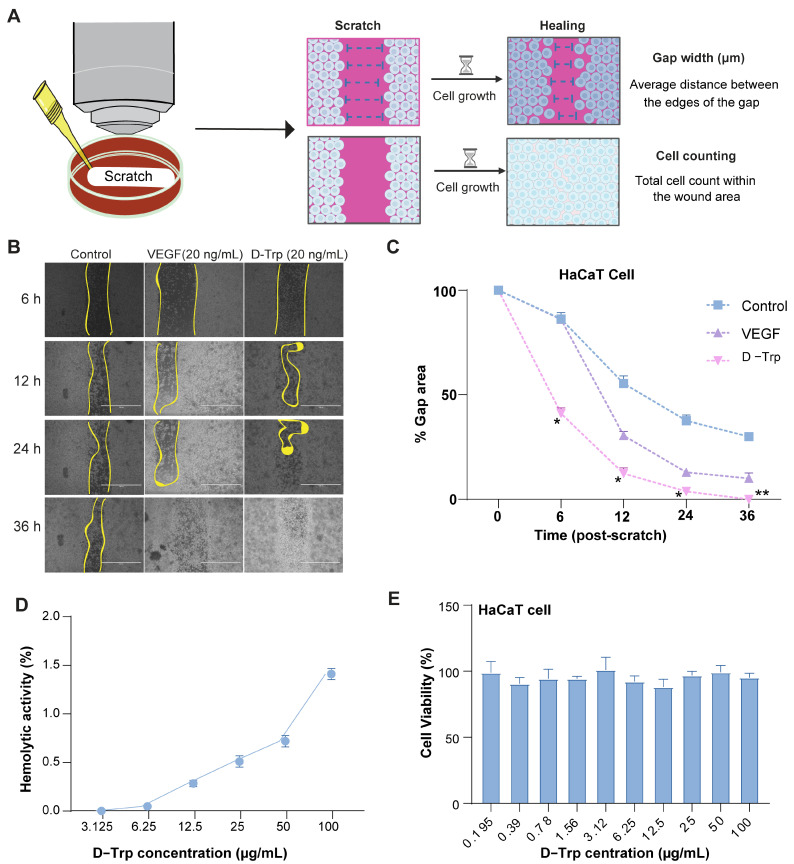
Effects of D-tryptophan on HaCaT cell migration. (**A**) Schematic figure of wound healing assay measurements. (**B**) The impact of D-Trp on HaCaT cell migration is illustrated at various time points, with a scale bar of 650 μm. (**C**) Quantification of the number of HaCaT cells that migrated after treatment (**D**) % hemolytic activity in human blood (**E**) % cell viability. Data are presented as mean ± standard error (SE) with a sample size of *n* = 3. Statistical significance is denoted as follows: * *p* < 0.05, ** *p* < 0.01.

To investigate the role of D-Trp in wound healing, we performed a wound healing assay using HaCaT cells, a well-established human keratinocyte cell line that is frequently used in wound healing research [32]. Figure 1A illustrates the cell migration process, detailing the key steps involved in the movement of cells from one location to another. This assay replicated a wound environment by introducing a linear scratch into a confluent monolayer of cells and subsequently monitoring the capacity of the cells to migrate and bridge the gap. The findings revealed that D-Trp treatment markedly augmented the migratory capacity of HaCaT cells compared to the control group (Figure 1B,C). Specifically, a marked increase in the rate at which D-Trp-treated cells migrated across the wound area was observed, effectively closing the gap more quickly than control cells, while amino acid treatment significantly accelerated wound closure, suggesting that D-Trp played a crucial role in promoting cell migration, a critical process for wound closure.

These findings strongly validated that D-Trp actively promoted cell migration, a key aspect of wound healing, further highlighting its potential therapeutic value in accelerating tissue repair. As illustrated in Figure 1D, the hemolysis rate of D-Trp at a concentration of 100 μg/mL was found to be less than 2%, indicating that D-Trp exhibited minimal hemolytic activity and is unlikely to induce rupture or lysis of human red blood cells under these conditions. Furthermore, Figure 1E confirmed that the cytotoxicity of D-Trp at the same concentration (100 μg/mL) was less than 1%, suggesting its negligible toxic effect on HaCaT cells at this concentration.

### 2.2. D-Trp Accelerates Wound Healing in a Non-Diabetic Model

**Figure 2:**
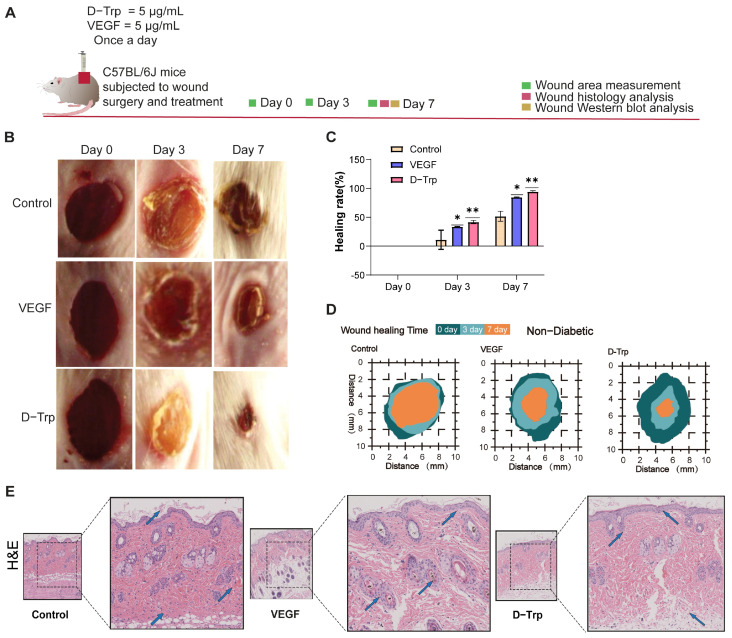
An evaluation of non-diabetic wound healing in vivo. (**A**) A schematic illustration of the treatment process alongside the progression of wound healing. (**B**) Quantification of wound healing processes on days 0, 3 and 7. (**C**) Representative images of chronic diabetic wounds that were treated with PBS, VEGF, and D-Trp. A schematic diagram in panel (**D**) further illustrates the percentage of wound closure for each group across the three time points. (**E**) Representative images of hematoxylin and eosin staining (H&E), which emphasize blood vessels, with blue arrows denoting newly formed vessels and hair follicles (scale bars: 100 μm and 20 μm). Data are expressed as mean ± standard error (SE) with a sample size of *n* = 5, and statistical significance is indicated as follows: * *p* < 0.05; ** *p* < 0.01.

Wound healing is a dynamic and multi-phase process that involves inflammation, tissue regeneration, and remodeling to restore skin integrity [33]. Effective wound healing depends on adequate epithelialization, granulation tissue formation, and a well-regulated inflammatory response [34]. In this study, we used a C57BL/6J mouse model with severe skin wounds. Figure 2A provides a schematic representation of the key processes involved in wound healing. The findings indicated that the D-Trp treatment group showed a notably faster wound healing rate compared to the control group (Figure 2B). Figure 2C illustrates the percentage of wounds that achieved complete closure over time, with the D-Trp group demonstrating a significantly higher proportion of fully healed wounds compared to controls. All experimental groups showed typical wound healing responses, including wound contraction and the formation of neo-tissue-supported wound beds. However, on days 3 and 7, the D-Trp group demonstrated a faster reduction in the wound area and an increase in tissue thickness compared to the control, as assessed by measuring the area of the injury (Figure 2D). In particular, on day 3, the D-Trp group exhibited substantial epithelial turnover and keratinization tissue, along with the appearance of new white hair, suggesting elevated epithelialization and hair follicle regeneration. On day 7, the D-Trp group was the first to develop a newly formed epidermis and surrounding hair growth, indicating more complete and accelerated wound closure. Histological analysis of skin sections using H&E staining revealed significant improvements in wound healing for the group treated with D-Trp relative to the control (Figure 2E).

Specifically, D-Trp treatment led to an increase in granulation tissue formation and epithelialization, along with a marked reduction in inflammatory cell infiltration at the wound site, as evidenced by H&E staining. These findings are consistent with previous studies that have demonstrated the role of tryptophan metabolites in improving wound healing, promoting tissue regeneration, and modulating the inflammatory response [35,36,37].

### 2.3. D-Trp Modulates Key Protein Expression Involved in Non-Wound Healing

**Figure 3:**
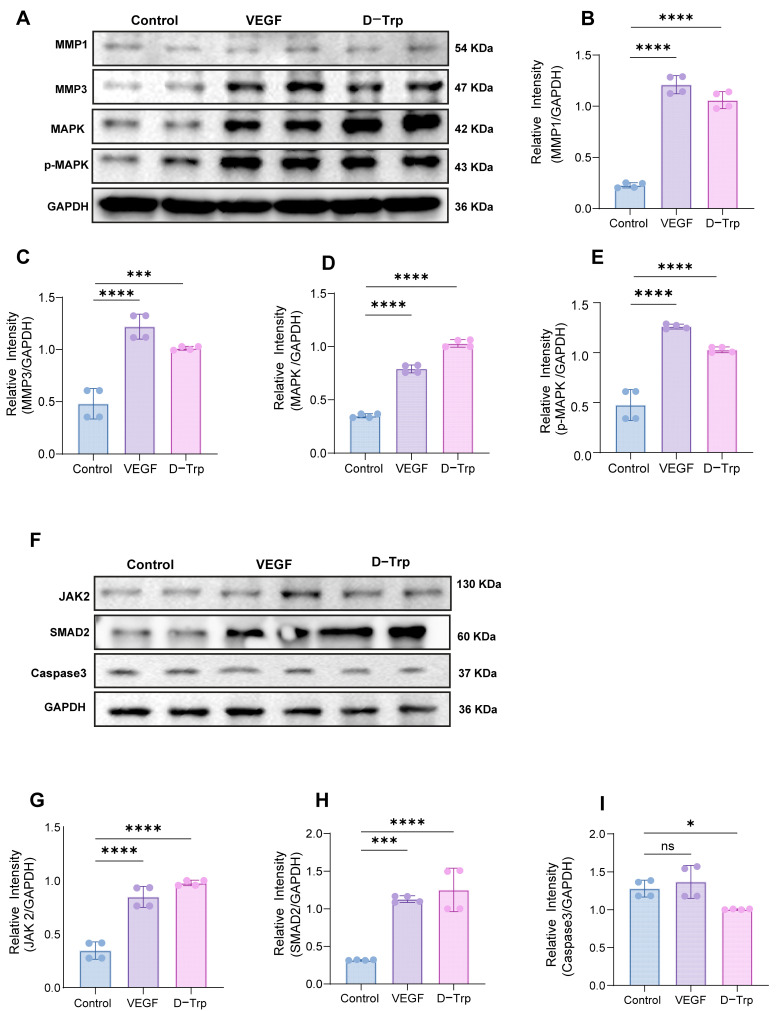
D-Trp regulates the expression of proteins associated with impaired wound healing. (**A**) Representative Western blot images of key proteins in skin wound tissues. (**B**–**E**) Quantitative analysis of MMP1, MMP3, MAPK, and phosphorylated MAPK (p-MAPK) expression levels, respectively. (**F**) Additional Western blot images of wound tissue samples. (**G**–**I**) Densitometric quantification of JAK2, SMAD2, and Caspase-3 protein levels in skin and tissues, respectively, from mice treated with phosphate-buffered saline (PBS), vascular endothelial growth factor (VEGF), or D-tryptophan (D-Trp). Data are presented as mean ± standard error (SE) (*n* = 4). Statistical significance is indicated as follows: ns (*p* > 0.05), * *p* < 0.05, *** *p* < 0.001, **** *p* < 0.0001.

The administration of D-Trp exerted significant modulatory effects on the expression of proteins implicated in wound healing (Figure 3). As shown in the representative Western blot images (Figure 3A), D-Trp treatment notably enhanced the levels of MMP1 and MMP3 (Figure 3B,C), indicative of improved extracellular matrix (ECM) remodeling through increased proteolytic activity [38]. Furthermore, D-Trp significantly elevated the expression of total MAPK and phosphorylated MAPK (p-MAPK) compared to control and VEGF-treated groups (Figure 3D,E). While p-MAPK is often normalized to total MAPK to evaluate pathway activation, in this analysis, both MAPK and p-MAPK were independently quantified relative to GAPDH. The observed parallel increase in both forms supports MAPK pathway involvement in promoting keratinocyte proliferation and tissue regeneration.

Additional Western blot analyses revealed marked upregulation of JAK2 (Figure 3G), highlighting potential activation of the JAK/STAT signaling pathway, which is central to inflammatory modulation and tissue repair [39]. Similarly, the expression of SMAD2 (Figure 3H) was increased in D-Trp-treated wounds, suggesting participation of the TGF-β/SMAD pathway in enhancing ECM deposition and wound contraction. Notably, caspase-3 levels were significantly reduced (Figure 3I), indicating a potent anti-apoptotic effect that may contribute to enhanced cellular survival in the wound bed [40]. Collectively, these results suggest that D-Trp promotes wound healing by modulating multiple molecular pathways involved in matrix remodeling, proliferation, and cell survival.

### 2.4. D-Trp Accelerates Wound Healing Diabetic Model

**Figure 4:**
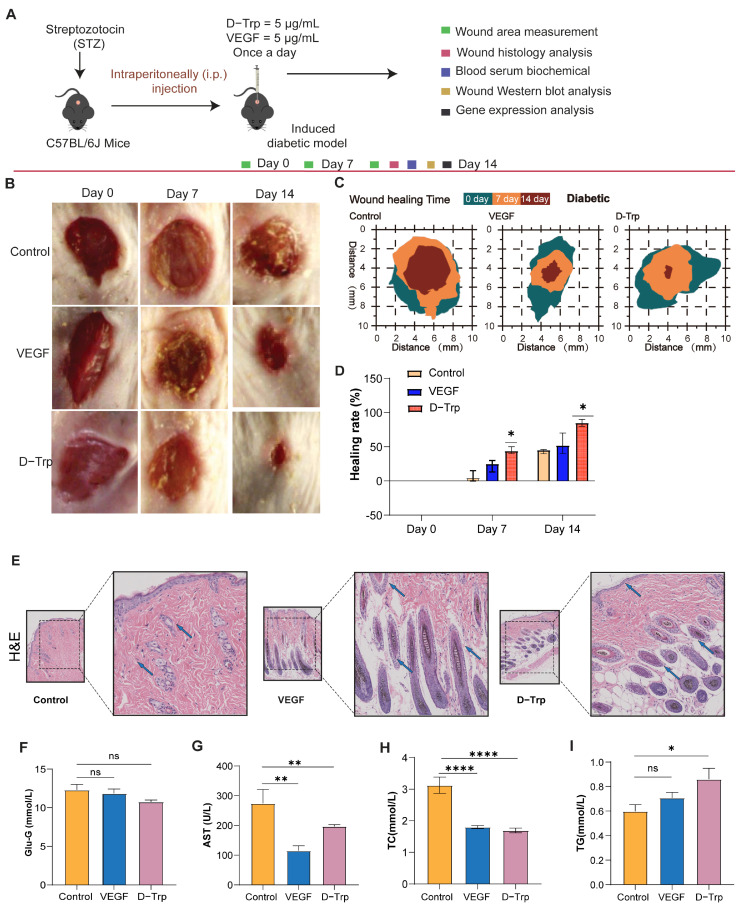
An evaluation of diabetic wound healing in vivo. (**A**) A schematic illustration of the treatment process alongside the progression of wound healing. (**B**) Wound healing processes at days 0, 7, and 14. (**C**) Representative images of chronic diabetic wounds that were treated with PBS, VEGF, and D-trp. (**D**) Schematic diagram illustrating the percentage of wound closure for each group at three different time points. (**E**) Representative images of hematoxylin and eosin (H&E) staining, highlighting blood vessels with blue arrows indicating newly formed vessels and hair follicles (scale bars: 100 μm and 20 μm). Analysis of biochemical plasma parameters following 14 days of intervention, (**F**) glucose levels, (**G**) aspartate aminotransferase (AST), (**H**) triglycerides (TG), and (**I**) total cholesterol (TC). Data are expressed as mean ± standard error (SE) with a sample size of *n* = 5, and statistical significance is indicated as follows: ns, *p* > 0.05; * *p* < 0.05; ** *p* < 0.01; **** *p* < 0.0001.

In an experimental framework of 14 days employing streptozotocin (STZ)-induced diabetic mice (Figure 4A), it was discovered that D-Trp significantly enhanced wound healing in this diabetic model at a concentration of 5 µg/mL, surpassing the efficacy observed in the control groups (Figure 4B). While all groups exhibited wound contraction and new tissue formation, the D-Trp group demonstrated a notably faster reduction in wound area and developed thicker, more robust tissue (Figure 4B), indicative of a more effective and comprehensive healing process. Furthermore, D-Trp-treated mice exhibited early signs of advanced epithelialization and tissue regeneration, marked by the collapse of newly formed tissue and the emergence of new hair growth (Figure 4C).

After 14 days, the D-Trp group showed the most significant healing response, with a fully restored epidermis and complete hair regrowth. These findings were consistent with existing research suggesting that tryptophan metabolites, including D-Trp, may play a role in promoting tissue repair and accelerating wound healing, particularly in diabetic conditions. Similar studies have demonstrated that amino acids like tryptophan enhance collagen deposition, fibroblast activity, and keratinocyte proliferation, all of which are crucial for effective wound closure and regeneration [41]. Moreover, the accelerated epithelialization and tissue regeneration observed in our study aligned with the findings of previous work on amino acid-based therapies that underscore their potential to improve wound healing in diabetic models [42,43]. Collectively, our results underscore the therapeutic potential of D-Trp as a promising candidate for enhancing wound healing outcomes in diabetic conditions.

### 2.5. D-Trp Modulates Key Protein Expression Involved in Diabetic Wound Healing

**Figure 5:**
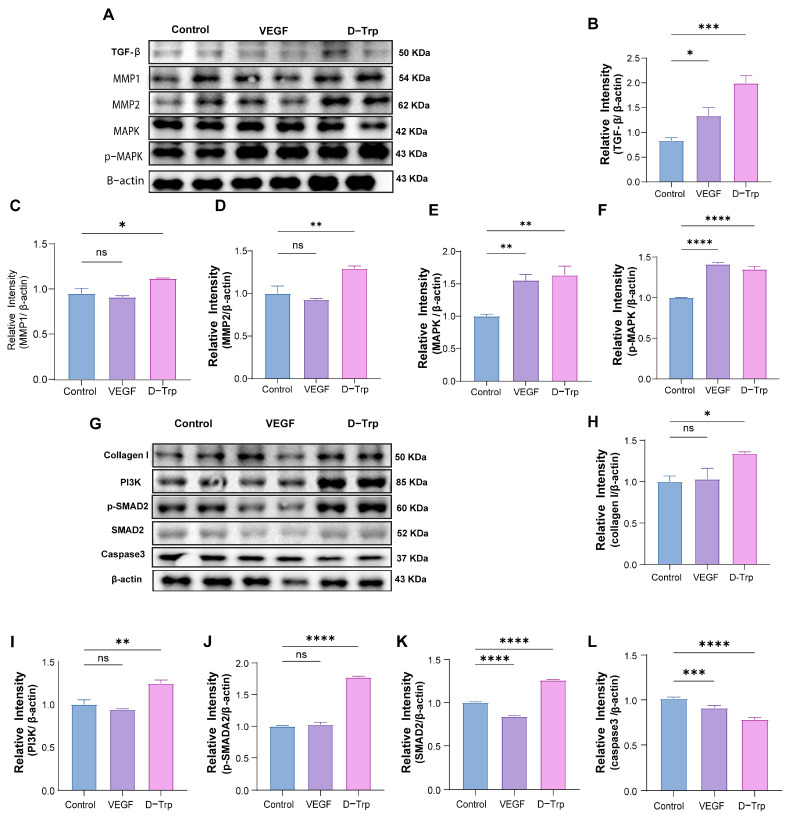
Western blotting analysis conducted in the diabetic wound model, 14 days post-surgery. (**A**) The intensity of Western blotting in skin wound samples. (**B**–**L**) A quantification of the results of Western blotting for various proteins, including TGF-β, MMP2, MAPK, phosphorylated MAPK (p-MAPK), JAK1, Collagen I, PI3K, phosphorylated SMAD2 (p-SMAD2), SMAD2, and Caspase-3, in skin samples from mice treated with PBS, VEGF, and D-Trp. The data are expressed as the mean ± standard error (SE) with a sample size of *n* = 4, and statistical significance is indicated as follows: ns, *p* > 0.05; * *p* < 0.05; ** *p* < 0.01; *** *p* < 0.001; **** *p* < 0.0001.

D-Trp treatment markedly improved wound healing in diabetic mice through coordinated activation of critical molecular pathways. Western blot analysis revealed a 2.39-fold upregulation of TGF-β (Figure 5B), indicating robust activation of this master regulator of fibrogenesis that stimulates fibroblast differentiation and collagen production. Concurrently, we observed a 1.18-fold increase in MMP1 and a 1.29-fold elevation in MMP2 (Figure 5C,D), demonstrating enhanced proteolytic capacity for ECM degradation and tissue remodeling essential for keratinocyte migration. Treatment also induced 1.63-fold-higher total MAPK levels with 1.35-fold-increased phosphorylation (Figure 5E,F), confirming activation of this central signaling cascade that coordinates cellular proliferation and migration through ERK1/2-dependent mechanisms [44].

Further analysis identified additional therapeutic mechanisms through which D-Trp enhances diabetic wound repair. The treated groups showed pronounced upregulation of collagen I (Figure 5H), reinforcing ECM stabilization through enhanced structural protein deposition, consistent with the observed TGF-β-mediated fibrogenic response. The concurrent elevation of PI3K (Figure 5I) and SMAD2 phosphorylation (Figure 5J,K) revealed dual activation of both immediate pro-survivals signaling and sustained TGF-β/SMAD transcriptional regulation [45], creating an optimal microenvironment for fibroblast proliferation and matrix production. Particularly significant was the dramatic suppression of Caspase-3 (Figure 5L), demonstrating D-Trp’s ability to counteract the excessive apoptosis that characterizes diabetic wounds [46].

These molecular changes work synergistically with D-Trp’s previously demonstrated upregulation of TGF-β, MMPs, and MAPK pathways, creating a comprehensive therapeutic effect that enhances wound healing through multiple coordinated mechanisms. The combined action of increased collagen I production and MMP-mediated ECM degradation establishes balanced tissue remodeling, while concurrent activation of PI3K, MAPK, and SMAD2 signaling pathways drives cellular repair processes. Furthermore, the significant reduction in Caspase-3 levels demonstrates potent inhibition of pathological apoptosis, preserving cellular viability in the wound environment. Together, these effects address the key pathological deficits in diabetic wound healing by simultaneously promoting ECM restructuring, activating cellular repair programs, and maintaining tissue viability through integrated molecular pathways.

### 2.6. D-Tryptophan Boosts Gene Expression to Promote Diabetic Wound Healing

**Figure 6:**
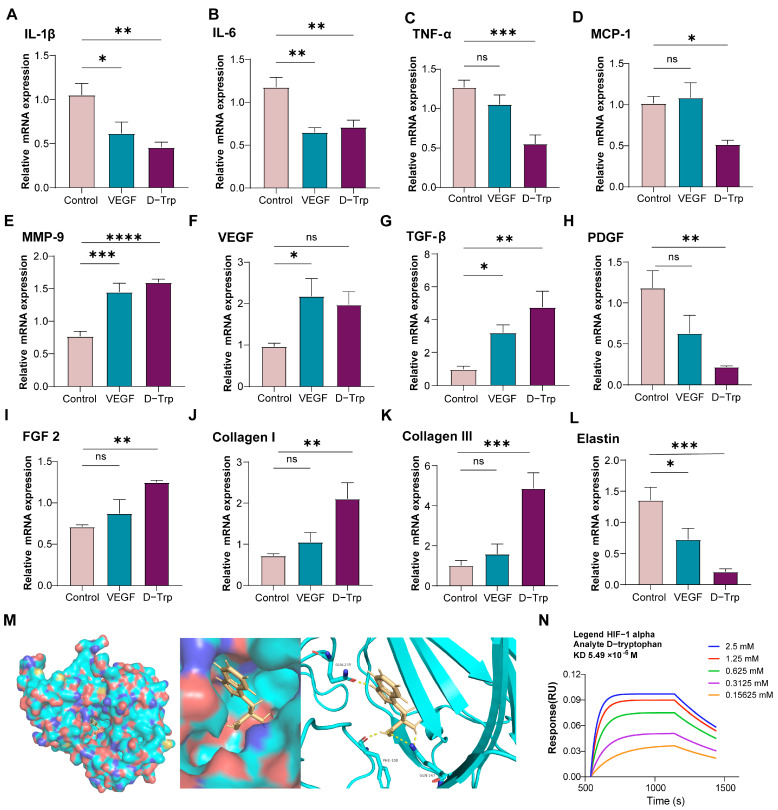
D-Trp stimulates the production of essential growth factors and ECM components, promoting fibroblast activity and ECM synthesis. (**A**–**L**) mRNA expression levels of *IL-1β*, *IL-6*, *TNF-α*, *MCP-1*, *MMP-9*, *VEGF*, *TGF-β*, *PDGF*, *FGF2*, *collagen I*, *collagen III* and *elastin*, respectively, in wound tissues were determined by RT-PCR. (**M**) Molecular docking analysis predicted a favorable binding interaction between D-Trp and hypoxia-inducible factor 1-alpha (HIF-1α). (**N**) The binding affinity between D-Trp and HIF-1α was assessed using the Octet^®^ BLI system. The data are expressed as the mean ± standard error (SE) with a sample size of *n* = 6, and statistical significance is indicated as follows: ns, *p* > 0.05; * *p* < 0.05; ** *p* < 0.01; *** *p* < 0.001; **** *p* < 0.0001.

Our investigation demonstrated that D-Trp administration significantly modulates gene expression profiles in diabetic wound models, exerting a potent anti-inflammatory effect. Notably, D-Trp treatment markedly downregulated key pro-inflammatory cytokines, including *IL-1β* (Figure 6A), *IL-6* (Figure 6B), *TNF-α* (Figure 6C), and *MCP-1* (Figure 6D). This suppression of inflammatory mediators highlights the therapeutic potential of D-Trp in attenuating excessive inflammation, a critical factor in facilitating efficient diabetic wound healing [47]. Concurrently, D-Trp treatment significantly upregulated *MMP-9* expression (Figure 6E), a critical mediator of extracellular matrix (ECM) degradation and remodeling, thereby promoting enhanced cellular migration and tissue regeneration [48].

Furthermore, D-Trp administration induced a marked increase in *VEGF* levels (Figure 6F), suggesting a robust pro-angiogenic response. This upregulation of *VEGF* underscores the potential of D-Trp to enhance neovascularization, a vital process for restoring nutrient and oxygen supply to the wound microenvironment and supporting effective tissue repair [49,50]. The significant upregulation of *TGF-β* (Figure 6G), *PDGF* (Figure 6H), and *FGF2* (Figure 6I), along with increased expression of *collagen I* (Figure 6J), *collagen III* (Figure 6K), and *elastin* (Figure 6L), demonstrates that D-Trp enhances the production of key growth factors and extracellular matrix (ECM) components. This coordinated upregulation suggests D-Trp promotes fibroblast activation, stimulates ECM synthesis, and drives tissue remodeling, ultimately accelerating wound closure and improving regenerative outcomes in diabetic wounds [51,52].

To elucidate the mechanistic basis of D-Trp-mediated wound healing, we investigated its interaction with hypoxia-inducible factor 1-alpha (HIF-1α), a master regulator of cellular responses to hypoxia [53]. Molecular docking analysis revealed a strong binding affinity between D-Trp and HIF-1α (Figure 6M), a finding subsequently validated by Bio-Layer Interferometry (BLI) using the Octet^®^ system (Figure 6N), which measured a binding affinity of 5.49 μM. Given HIF-1α’s pivotal role in orchestrating angiogenesis, cell proliferation, and tissue regeneration under hypoxic conditions, a hall-mark of wound microenvironments, these results suggest that D-Trp exerts its therapeutic effects through direct modulation of HIF-1α activity [54,55]. This mechanistic framework is further supported by our observation of upregulated *VEGF* expression and other wound-healing genes, collectively indicating that D-Trp enhances tissue repair, at least partially, via HIF-1α-dependent pathways.

## 3. Discussion

This study establishes D-Trp as a promising therapeutic candidate for diabetic wound healing by addressing multiple pathological deficits characteristic of impaired healing. Diabetic wounds typically demonstrate compromised repair due to diminished fibroblast activity, insufficient collagen production, and chronic inflammation [10,56]. The enhanced wound healing observed in the D-Trp group suggested a multifaceted role for D-Trp in promoting tissue repair. The faster wound area shrinkage and thicker wound tissue observed in the D-Trp group pointed towards increased fibroblast activity and collagen deposition. This was further supported by the early development of a newborn epidermis and structures resembling hair follicles in the D-Trp group, suggesting that D-Trp may promote epithelial cell proliferation and differentiation, thereby contributing to re-epithelialization and the restoration of skin integrity. Although hair follicle regeneration on mature scars is generally limited, the presence of hair follicle-like structures in our model highlights the potential of D-Trp to stimulate aspects of skin repair beyond simple wound closure. Further studies are needed to confirm whether these structures can fully mature and restore hair growth in scar tissue.

The coordinated upregulation of MMP1 and MMP3 demonstrates D-Trp’s capacity to enhance ECM proteolytic activity, facilitating the tissue remodeling essential for proper wound regeneration [57,58,59,60]. The concurrent activation of MAPK signaling is critical in modulating cellular processes, including proliferation, differentiation, and inflammatory responses, all of which are vital for tissue repair [44], suggesting that D-Trp may contribute to enhanced cell signaling and potentially improved tissue repair. The upregulation of SMAD2, a key transcription factor involved in TGF-β signaling, supports the notion that D-Trp may enhance TGF-β signaling, a crucial pathway for wound healing and ECM synthesis [61]. The downregulation of caspase-3, a critical executioner caspase in apoptosis, indicated that D-Trp may protect cells from programmed cell death, contributing to a more favorable healing environment [62].

The intricate relationship between gene expression and wound healing is pivotal in understanding the mechanisms that govern tissue repair [63]. The observed gene expression changes, combined with our binding affinity data, provide compelling evidence for the complicated influence of D-Trp on wound healing, highlighting its potential as a therapeutic agent. The downregulation of pro-inflammatory cytokines indicated a potent anti-inflammatory effect of D-Trp, mitigating the detrimental effects of excessive inflammation that can hinder tissue repair and increase infection susceptibility. Furthermore, the upregulation of MMP-9 and VEGF, coupled with the direct interaction between D-Trp and HIF-1α, suggested that D-Trp promoted efficient ECM remodeling, angiogenesis, and likely played a role in activating HIF-1α, enhancing its pro-angiogenic effects. The increased expression of key growth factors and ECM components further supported its ability to stimulate fibroblast activity, enhance ECM synthesis, and ultimately contribute to improved tissue regeneration and wound closure. These findings, particularly the observed effects in both diabetic and non-diabetic models, suggest that D-Trp might offer a promising therapeutic strategy for promoting wound healing, especially in individuals with impaired healing, such as those with diabetes, who often experience delayed healing and complications.

While VEGF is a well-characterized pro-angiogenic factor that directly stimulates vascular endothelial cell proliferation and new blood vessel formation, D-Trp also modulates epithelial cell proliferation and inflammatory responses, potentially enhancing multiple facets of tissue repair. Moreover, as a small molecule amino acid, D-Trp offers advantages in stability, ease of synthesis, and cost-effectiveness compared to protein-based therapeutics like VEGF, which require complex production and storage. These features suggest that D-Trp could serve as a more practical and accessible therapeutic agent for wound healing applications.

In conclusion, D-Trp influences protein expression related to wound healing by upregulating matrix metalloproteinases (MMPs), activating MAPK and JAK signaling pathways, and downregulating caspase-3. Additionally, it reduces inflammation, enhances extracellular matrix (ECM) turnover, stimulated angiogenesis via HIF-1α activation, and promoted ECM synthesis. Our study offers a promising strategy, especially in diabetic wound care, as well as enlarging the clinical approaches and documental research on amino acid biology in chronic diseases.

### Limitation of This Study

One limitation of this study is the absence of pharmacokinetic or tissue distribution data to evaluate the local retention and potential systemic absorption of D-Trp following topical application. Although no overt signs of local irritation or adverse skin reactions were observed during treatment, a formal assessment of skin tolerance particularly in diabetic models was not performed. Additionally, only a single concentration of D-Trp was tested, selected for comparison with VEGF, and a dose–response evaluation was not conducted in order to minimize animal use. Moreover, the study focused on early to mid-stage wound healing and did not assess long-term outcomes such as scar formation, tissue remodeling, or mechanical integrity of the regenerated skin. Future studies should address these aspects to comprehensively evaluate the safety, efficacy, and clinical translational potential of D-Trp in wound healing.

## 4. Materials and Methods

### 4.1. In Vitro Wound Healing Assay

We carried out a wound healing assay as previously described [64]. Briefly, HaCaT human keratinocyte cells (catalog number M-C1056) were obtained from the Kunming Cell Bank, Kunming Institute of Zoology, Chinese Academy of Sciences, Kunming, Yunnan, China, and were seeded in 12-well plates at a density of 2 × 10^5^ cells/well. Once the cells reached 80–90% confluency, the medium (DMEM, Corning, Corning, NY, USA; #10-092-CVRC) was replaced with fresh medium containing either no supplement (Control), VEGF (20 ng/mL, MedChemExpress LLC, Monmouth Junction, NJ, USA, HY-P70458A), or D-tryptophan (D-Trp, 20 ng/mL, MedChemExpress LLC, Monmouth Junction, NJ, USA; #HY-W012479). Two hours after treatment, a scratch wound was introduced by scraping the cell monolayer with the tip of a sterile pipette. Cell migration was assessed by measuring wound closure distances using ImageJ software (version 1.47v; National Institutes of Health, Bethesda, MD, USA). Images were captured at 6, 12, 24, and 36 h using an Olympus CX41 microscope (Olympus, Tokyo, Japan). All experiments were performed in triplicate.

### 4.2. Hemolysis Analysis

Whole blood was collected from healthy mice and immediately mixed with an anticoagulant solution. The anticoagulated blood was subsequently washed twice with sterile normal saline (0.9% NaCl) under aseptic conditions. Following centrifugation (300× *g*, 10 min), the cell pellet was resuspended in the appropriate buffer to achieve a final concentration of 1 × 10^7^ to 1 × 10^8^ cells/mL. The diluted red blood cell suspension is mixed with a D-Trp sample dissolved in normal saline. The mixture was incubated at 37 °C for a duration of 30 min, subsequent to which it was subjected to centrifugation at 1000 rpm for 5 min. The resultant supernatant was then analyzed at a wavelength of 540 nm. The negative control was treated with normal saline, and the positive control was treated with Triton X-100, and the percentage of hemolysis was calculated as follows: percentage of hemolysis H% = (sample A − negative control)/100% × positive control.

### 4.3. Cytotoxicity Analysis

The cell counting kit 8 (CCK-8 MedChemExpress LLC, Monmouth Junction, NJ, USA; #HY-KO301) assay was used to determine cytotoxicity on the HaCaT cell line (Kunming cell bank). The cells were maintained in an appropriate growth medium until they reached 70% to 80% confluency within the tissue culture flask. Subsequently, the cells were detached using trypsin and enumerated with a hemocytometer. There were approximately 1 × 10^4^ to 1 × 10^5^ cells per well. After 24 h of incubation to allow cell adherence, the culture medium was replaced with serial dilutions of D-Trp prepared in growth medium, ranging from 0.195 to 100 µg/mL. After cell adhesion, D-Trp-treated serial dilutions in growth medium were prepared and the medium in the wells was replaced with these dilutions. Control wells containing untreated cells were set up for comparison with control wells containing known cytotoxic agents. The plates were incubated at 37 °C for 24 h, with 5% CO_2_. Following the incubation period, the CCK-8 solution was introduced to each well according to the manufacturer’s guidelines and further incubated for 1~4 h to promote the colorimetric reaction. Subsequently, the absorbance at the wavelength of 450 nm was determined utilizing a microplate reader. Cell viability is quantified as a percentage, employing the following formula: cell viability (%) = (A sample/A control) × 100%, where sample A represents the absorbance of treated cells and control A represents the absorbance of untreated control cells. The cytotoxic effect of D-Trp on HaCaT cells was evaluated by the analysis results.

### 4.4. Binding Affinity

We used the Octet^®^ Bio-Layer Interferometry (BLI) system (Sartorius AG, Göttingen, Germany) to detect the binding affinity of D-Trp to HIF-1α protein, MCE #HY-P74888. We prepared the assay buffer and the reference buffer. Initially, D-Trp solutions were prepared at concentrations ranging from 2.5 mM to 0.15625 mM. Subsequently, recombinant HIF-1α protein (MedChemExpress, Monmouth Junction, NJ, USA) was biotinylated using a reagent from Thermo Fisher Scientific (Waltham, MA, USA) and diluted to a concentration of 10 µg/mL to facilitate its immobilization. The biosensors were then loaded with streptavidin (Sartorius AG, Göttingen, Germany). Subsequently, the Octet^®^ BLI system was utilized to load streptavidin onto SA biosensors according to the manufacturer’s protocol. The biosensors, loaded with streptavidin, were then allowed to incubate in the HIF-1α solution for a sufficient duration to facilitate receptor binding to streptavidin. Following binding, the biosensors were rinsed using assay buffer. Subsequently, the biosensors were placed in the reference buffer for a predetermined duration to establish a baseline signal. Thereafter, the biosensors were transferred to solutions containing varying concentrations of D-Trp to facilitate binding of the ligand to the immobilized HIF-1α for a period of 300 s. The biosensors were then returned to the reference buffer to observe the dissociation of D-Trp from the HIF-1α over a span of 150 s. The experiment incorporated appropriate controls, including biosensors containing only streptavidin (without the receptor) or biosensors with a receptor but without the ligand.

### 4.5. Molecular Docking

Molecular docking simulations were conducted using AutoDock Vina (version 1.1.2). The three-dimensional structure of human HIF-1α was retrieved from the Protein Data Bank (PDB ID: 4H6J), while the 3D structure of D-Trp was obtained from the PubChem database (CID: 9060). Docking was performed to predict the binding interactions between HIF-1α and D-Trp, and the corresponding binding energies were calculated by AutoDock Vina. Visualization and structural analysis of the docking results were carried out using PyMOL (version 2.5).

### 4.6. Ethical Considerations

This study adhered to regulatory compliance that involved ethical regulations and guidelines related to human and animal research. This included protecting the confidentiality of the subjects, obtaining informed consent, and ensuring ethical and humane treatment of the animals throughout the study.

### 4.7. Animal Subjects

The experimental animal protocols received approval from the Institutional Animal Care and Use Committee (IACUC) of the Kunming Institute of Zoology, Chinese Academy of Sciences, under the approval number IACUC-RE-2024-08-013. The handling and housing of the animals adhered to the guidelines established by the Animal Experimental Center of the Kunming Institute of Zoology. Mice were purchased at the age of 8 weeks from Charles River Laboratory Animal Technology Co., Ltd. (Wuhan, China), and were subsequently housed in groups under controlled environmental conditions. The mice were kept on a 12 h light–dark cycle in a temperature range of 22 °C to 24 °C. They received ad libitum access to standard laboratory food and water, except during specified periods for glucose level testing, during which their food access was restricted.

### 4.8. Induction of Diabetes

Experimental diabetes was established in mice via a single intraperitoneal injection of streptozotocin (STZ, YEASEN Biotechnology, Shanghai, China; #60256ES80) at 80 mg/kg body weight. Glycemic status was assessed hourly using a glucometer. Mice were classified as diabetic if their blood glucose concentrations consistently exceeded 11.1 mmol/L for at least 3 consecutive days.

### 4.9. Treatment Groups and Administrations

To evaluate the therapeutic potential of D-Trp in promoting wound healing, full-thickness dorsal wounds of 6 mm diameter were created in C57BL/6J mice. A total of 30 male mice aged 10 to 12 weeks were used, including 15 healthy controls and 15 with experimentally induced diabetes. The mice were randomly assigned into three groups of five animals each: control (topical PBS, Corning #21-040-CV), VEGF, or D-Trp treatment. Following a one-week acclimatization period, the mice were anesthetized by intraperitoneal injection of 2% pentobarbital (Cayman Chemical Company, Ann Arbor, MI, USA; #76-74-4) at a dose of 4 to 4.5 µL per 20 g of body weight. The fur on the dorsal region, specifically between the tail and back, was removed using Veet^®^ Pure Hair Removal Cream (Reckitt Benckiser, Rueil-Malmaison, France) to prepare the surgical site under aseptic conditions. Full-thickness wounds were then generated using a 6 mm biopsy punch. After wounding, treatments were applied topically once daily using a pipette. The control group received 30 µL of PBS, while the VEGF and D-Trp groups each received 30 µL of their respective treatments at a concentration of 5 µg/mL. No occlusive dressing was applied to the wounds. Wound area measurements and tissue sample collections were conducted at different time points to account for the distinct healing rates in the two mouse models. For normal mice, wounds were assessed on days 1, 3, and 7 post-wounding, with tissue samples collected on day 7. For diabetic mice, wounds were evaluated on days 1, 7, and 14, with tissue harvested on day 14. This extended timeline for diabetic mice reflects their delayed wound healing compared to normal mice, allowing for a more accurate assessment of treatment effects. Throughout the study, wounds were monitored daily for signs of infection or other complications. Wound sites were photographed using an Olympus E-M10 digital camera, and wound boundaries were traced and measured using ImageJ software.

### 4.10. Wound Healing Assessment

The percentage of wound closure was calculated at each time point using the following formula: wound closure (%) = [(baseline wound area − current wound area)/baseline wound area] × 100.

### 4.11. Sample Collection for Histology, Western Blot, and RNA Analysis

At the designated endpoints, skin tissue surrounding the wound site was collected from five mice per group. Approximately 5–10 mm^2^ of full-thickness skin was harvested from each mouse. The collected tissue was divided into three portions for downstream analysis. One portion was fixed in 4% paraformaldehyde and processed for histological evaluation. A second portion was snap-frozen in liquid nitrogen and stored at −80 °C for subsequent protein extraction and Western blot analysis. The remaining portion was immediately processed for total RNA extraction and gene expression analysis.

### 4.12. Histological Analysis

At the experimental endpoint, the mice were euthanized and wound tissues collected for histological examination. Standard tissue processing included fixation in 4% neutral-buffered formalin, paraffin embedding, and sectioning at 5 µm thickness. Hematoxylin and eosin (H&E) staining was performed to assess general tissue morphology.

### 4.13. Western Blot

Western blot analysis was used to assess protein expression in wound tissues. Proteins were extracted from the wound sites, separated on 12% SDS-PAGE gels, and transferred onto polyvinylidene fluoride (PVDF) membranes. Membranes were blocked with 5% bovine serum albumin (BSA) for 1 h at room temperature, followed by overnight incubation at 4 °C with primary antibodies against collagen I abcam #ab6586, TGF-β Zen-Bio.cn #R22797, MMP1 abcam #ab51074, MMP2 abcam #ab92536, MMP3 abcam #ab52915, MAPK Zen-Bio.cn #R10024, phosphorylated MAPK (P-MAPK) Zen-Bio.cn #310289, JAK2 Zen-Bio.cn #R24775, PI3K abcam #ab283852, phosphorylated SMAD2 (P-SMAD2) abcam #ab2808888, SMAD2 abcam #ab63176, and Caspase-3 abcam (all at 1:1000 dilution), while GAPDH affinity #AF7021 and β-actin Zen-Bio.cn #250132, 1:10,000. After washing, membranes were incubated with HRP-conjugated secondary antibodies for 1 h at room temperature. Immunoreactive bands were detected using the ImageQuant LAS 4000 mini-imaging system (GE Healthcare, Little Chalfont, Buckinghamshire, UK).

### 4.14. RNA Extraction and Quantitative Real-Time PCR Analysis

Total RNA was extracted from wound tissues using the TRIzol™ Reagent (Invitrogen, Thermo Fisher Scientific, Waltham, MA, USA; Cat# 15596018) according to the manufacturer’s protocol. To quantify the expression levels of genes such as TNF-α, IL-1β, IL-6, MCP-1, MMP-9, VEGF, TGF-β, PDGF, FGF 2, collagen I, collagen III, and elastin, quantitative real-time PCR (qPCR) was performed. A total of 500 ng of RNA was reverse transcribed into cDNA using the 5× All-in-One MasterMix Kit (ABM, Applied Biological Materials Inc., Richmond, BC, Canada; Cat# G492), following the manufacturer’s instructions. The qPCR reactions were carried out in 20 μL volume using the BlasTaq 2× qPCR MasterMix (Abm, Canada; q#G892) in conjunction with the Step One Plus Real-Time PCR system (Thermo, Waltham, MA, USA). The expression levels of the target genes were normalized to those of GAPDH mRNA, which served as an internal control. The primer sequences used for qPCR are provided in Table 1.

### 4.15. Statistical Analysis

Data were analyzed using GraphPad Prism version 10.1.2. All results are expressed as mean ± standard deviation (SD). Sample sizes varied between assays due to differences in tissue availability and specific methodological requirements; however, all sample sizes were sufficient to ensure robust statistical analysis. For comparisons between two groups, an unpaired two-tailed Student’s *t*-test was used. For comparisons involving more than two groups, one-way analysis of variance (ANOVA) followed by Tukey’s post hoc test was performed. A *p*-value of <0.05 was considered statistically significant.

## Figures and Tables

**Table 1:** Primer sequences devised for RT-PCR analysis.

Gene	Forward (5′ → 3′)	Reverse (5′ → 3′)
*GAPDH*	GAAGGTCGGTGTGAACGGAT	AATCTCCACTTTGCCACTGC
*TNF-α*	TCTTCTCATTCCTGCTTGTGG	GGTCTGGGCCATAGAACTGA
*IL-1β*	GCAACTGTTCCTGAACTCAACT	ATCTTTTGGGGTCCGTCAACT
*IL-6*	TAGTCCTTCCTACCCCAATTTCC	TTGGTCCTTAGCCACTCCTTC
*MCP-1*	TTAAAAACCTGGATCGGAACCAA	GCATTAGCTTCAGATTTACGGGT
*MMP-9*	CGTCGTGATCCCCACTTACT	AACACACAGGGTTTGCCTTC
*VEGF*	GCACATAGAGAGAATGAGCTTCC	CTCCGCTCTGAACAAGGCT
*TGF-β*	CTCCCGTGGCTTCTAGTGC	GCCTTAGTTTGGACAGGATCTG
*PDGF*	CGGCCTGTGACTAGAAGAGG	GGGTCACTTCACACTTGCAT
*FGF 2*	CACCCTCACATCAAGCTACAACTTCA	TCAGCTCTTAGCAGACATTGGAAGA
*Collagen I*	GCTCCTCTTAGGGGCCACT	CCACGTCTCACCATTGGGG
*Collagen III*	CTGTAACATGGAAACTGGGGAAA	CCATAGCTGAACTGAAAACCACC
*Elastin*	TGCCTGGAGACATTTCCCTAG	GGTGCTCCAACATTTCCCAT

## Data Availability

The data that support the findings of this study are available from the corresponding author upon reasonable request.

## Abbreviations

The following abbreviations are used in this manuscript:

DFUs	diabetic foot ulcers
PBS	phosphate-buffered Saline
VEGF	vascular endothelial growth factor
SMAD2	mothers against decapentaplegic homolog 2
MMP	matrix metalloproteinases
ECM	extracellular matrix
MAPK	mitogen-activated protein kinase
HIF-1α	hypoxia-inducible factor 1-alpha
RT-PCR	reverse transcription-polymerase chain reaction
GAPDH	glyceraldehyde 3-phosphate dehydrogenase
TNF-α	tumor necrosis factor-alpha
IL-1β	interleukin-1 beta
IL-6	interleukin-6
MCP-1	monocyte chemoattractant protein-1
TGF-β	transforming growth factor-beta
PDGF	platelet-derived growth factor
FGF 2	fibroblast growth factor
